# The Variety of Carbon-Metal Bonds inside Cu-ZSM-5 Zeolites: A Density Functional Theory Study

**DOI:** 10.3390/ma3042516

**Published:** 2010-04-05

**Authors:** Takashi Yumura, Saki Hasegawa, Atsushi Itadani, Hisayoshi Kobayashi, Yasushige Kuroda

**Affiliations:** 1 Department of Chemistry and Materials Technology, Kyoto Institute of Technology, Matsugasaki, Sakyo-ku, Kyoto, 606-8585, Japan; 2 Department of Fundamental Material Science, Division of Molecular and Material Science, Graduate School of Natural Science and Technology, Okayama University, Tsushima, Kita-ku, Okayama 700-8530, Japan

**Keywords:** density functional theory calculation, orbital interactions, molecular symmetry, vibration, copper, the restricted environment of a zeolite host

## Abstract

Large-scale density functional theory calculations (DFT) found various types of binding of an unsaturated hydrocarbon (C_2_H_2_ and C_2_H_4_) to a ZSM-5 zeolite extraframework copper cation. We employed the DFT calculations based on the B3LYP functional to obtain local minima of an unsaturated hydrocarbon adsorbed on one or two copper cations embedded inside ZSM-5, and then compared their stabilization energies. The DFT results show that the stabilization energies are strongly dependent on the copper coordination environment as well as configurations of two copper cations. Consequently, the inner copper-carbon bonds are influenced substantially by a nanometer-scale cavity of ZSM-5.

## 1. Introduction

Interactions between a transition metal atom and a hydrocarbon have attracted many researchers [1,2,3,4,5,6,7,8,9], because the interactions may result in an activation of the hydrocarbon. The interactions weaken the C–H and C–C bonds of a hydrocarbon, and therefore facilitate the transformation of a hydrocarbon into a more valuable species. The activation of the C–H and C–C bonds is a key in catalytic reactions in heterogeneous and homogeneous systems. Thus we need to obtain detailed information on the activation processes for the purpose of constructing a promising catalyst. One of the well-known examples is that a transition metal atom binds coordinatively to an unsaturated hydrocarbon, such as alkenes and alkynes, called Dewar-Chatt-Duncanson models [10]. In these models, *π* and π* orbitals of an unsaturated hydrocarbon are responsible for the interactions with a transition metal cation, because these orbitals match *d* orbitals in terms of orbital symmetry. Such orbital interactions can result in electron transfers between the two. If *π* orbitals of an unsaturated hydrocarbon are depopulated through the interactions with a transition metal atom, or its π* orbitals are populated, the CC bonds of unsaturated hydrocarbons are activated.

Of course the electron transfers depend on types of transition metal atom as well as its coordination environment [10]. When a transition metal atom is embedded in a nanometer-scale cavity of a host, the interactions with a guest unsaturated hydrocarbon can be further affected by host confinement. The confinement effects on the inner bond formation have been well discussed in our recent theoretical studies [11,12,13,14,15,16]. In particular, we found various types of binding of a guest molecule into copper cations enclosed in the restricted environment of a ZSM-5 zeolite [14,15,16]. Thus it is intriguing to investigate whether the zeolite confinement can have an impact on its inner catalytic reactions. Along our previous studies, we have a special interest on how a guest molecule interacts with an extraframework copper cation of the ZSM-5 zeolite, because Cu–ZSM-5 exhibits unique catalytic behaviors [17]. With respect to chemical phenomena involving unsaturated hydrocarbons inside copper-containing zeolites, they can afford to catalyze the formation of diynes from alkyenes [18] and the partial oxidization of propylene into acrolein [19]. In these catalytic reactions, alkynes and alkenes are expected to coordinate to embedded copper cations. However, our knowledge how the zeolite confinement affects the inner copper–carbon bond formation is still lacking. The clarification will contribute to construct catalysts that can form selectively a desirable product.

**Figure 1:**
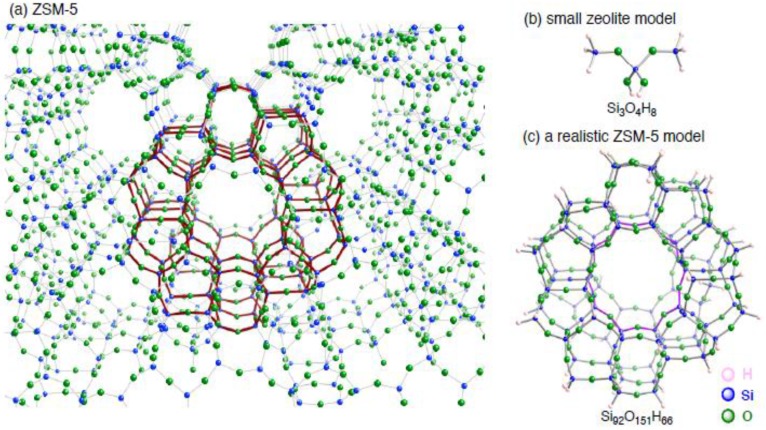
(a) The structures of ZSM-5 zeolite, models of aluminum-free ZSM-5, (b) Si_3_O_4_H_8_ and (c) Si_92_O_151_H_66_. The Si_92_O_151_H_66_ model corresponds to the red part in ZSM-5, and terminal atoms are saturated by H atoms.

In this direction, one of the promising tools is computer simulations based on quantum chemistry, in particular density functional theory (DFT) methods, because DFT results can provide atomic-scale view of the metal–carbon bond formation inside a zeolite cavity. Accordingly we employed DFT calculations to analyze how the restricted environment affects the inner coordination bonds. In this study, we focus on copper–carbon bonds formed inside a nanometer-sized cavity surrounded by a ten-membered ring of copper-exchanged ZSM-5 (Cu–ZSM-5) in Figure 1. In the present study, we will discuss two issues: (a) how an unsaturated hydrocarbon (acethylene or ethylene) interacts with an extraframework copper cation of ZSM-5, and (b) factors determining characters of copper–carbon bonds formed in the restricted environment of a ten-membered ring of ZSM-5.

## 2. Computational Section

In order to investigate interactions between an unsaturated hydrocarbon (acethylene or ethylene) and an extraframework copper cation of ZSM-5, we employed a hybrid Hartree–Fock/DFT method (B3LYP) [20,21,22,23,24], in the Gaussian 03 program package [25]. ZSM-5 zeolite consists of 5- and 6-membered rings (MRs) on channel walls, and 10-MRs in the straight and sinusoidal channels, as shown in Figure 1. Note that the 10-MRs are on the order of nanometers in terms of separation between diametrically-opposed Si atoms. In this study we adopt Si_3_O_4_H_8_ and Si_92_O_151_H_66_ clusters as models of aluminum-free ZSM-5 (silicalite) [26], as shown in the right-hand side of Figure 1. The Si_92_O_151_H_66_ model, whose terminal Si atoms are bound by H atoms, corresponds to the red part of the ZSM-5 framework [26], and contains ZSM-5 ten-MRs explicitly. The B3LYP calculation shows that the model has purple ten-MR cavities whose diametrically-opposed Si atoms are ~9.4 Å apart [14,15]. The cavity sizes are essentially identical to those observed experimentally. Thus the model is realistic to represent a 10-MR cavity of ZSM-5.

Using the realistic Si_92_O_151_H_66_ model in Figure 1, we constructed a Cu*_n_*–ZSM-5 model (*n* = 1 or 2) where a copper cation is located in the vicinity of an aluminum atom substituted for a silicon atom within the purple ten-MR. To obtain optimized geometries for an unsaturated hydrocarbon adsorbed on a copper active center inside the ZSM-5 model, we used the 6-311G* basis set for the adsorbing molecule and the Cu^+^ cations [27,28,29], and the 6-31G* basis set for substituted Al atom and the two O atoms bound to the substituted Al atom, and are usually coordinated by the cations [30,31,32], and the 3-21G basis set for other atoms in the zeolite framework [33,34,35]. After the optimizations for an unsaturated hydrocarbon adsorbed on Cu*_n_*–ZSM-5, we estimated their stabilization energy *E*_stabilization_, defined as *E*(adsorbent*–*Cu*_n_*–ZSM-5) – *E*(adsorbent) – *E*(Cu*_n_*–ZSM-5), where *E*(adsorbent*–*Cu*_n_*–ZSM-5) is the total energy of an optimized structure for an adsorbent bound to Cu*_n_*–ZSM-5, *E*(adsorbent) is that of an optimized structure for an adsorbent, and *E*(Cu*_n_*–ZSM-5) is that of an optimized structure for Cu*_n_*–ZSM-5. In the estimation, the *E*_stabilization_ values were corrected for basis set superposition errors (BSSEs) by using the counterpoise method [36]. Within the B3LYP calculations, the optimized separations between a copper cation and a framework oxygen atom fall in the range of 1.89 to 2.55 Å, being consistent with the previous DFT results [37,38,39,40,41,42,43,44]. The ranges of the calculated Cu–O separations are in good agreement with those obtained from the XRD [45] and EXAFS [46,47,48] analyses (1.98~2.56 Å). Thus the theoretical method of our choice is appropriate for the present study.

## 3. Results and Discussion

### 3.1. ZSM-5 containing monocopper cation (Cu_1_–ZSM-5)

#### 3.1.1. The small monocopper zeolite model

**Figure 2:**
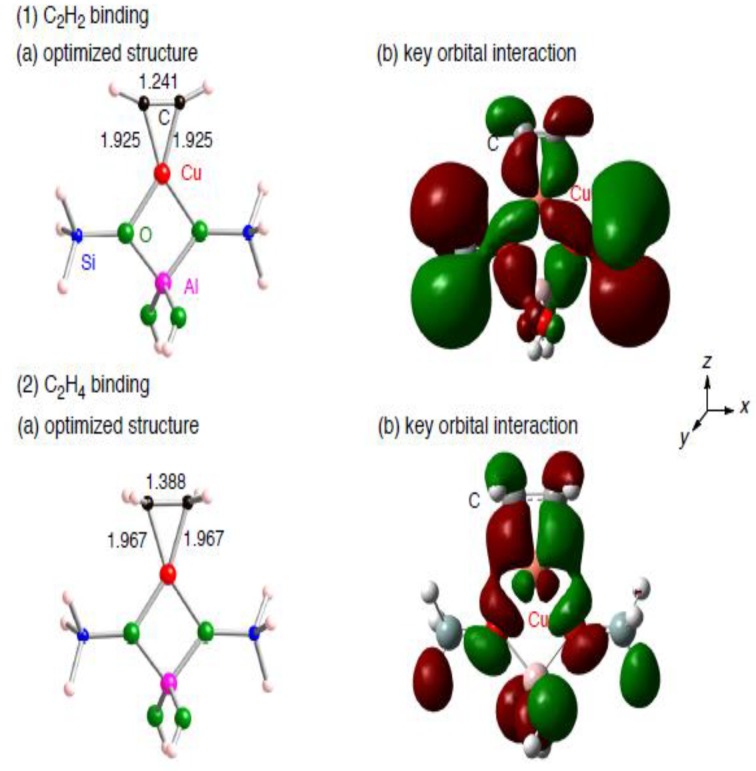
(a) Optimized structures for the binding of an unsaturated hydrocarbon ((1) C_2_H_2_ or (2) C_2_H_4_) into the monocopper cation bound to the AlSi_2_O_4_H_8_ model, and (b) their key orbital interactions.

First we investigate bindings of an unsaturated hydrocarbon into a monocopper cation bound to a small zeolite framework (AlSi_2_O_4_H_8_) for obtaining a baseline for comparison. In the small zeolite model, the monocopper cation is coordinated by two framework oxygen atoms. Using the small zeolite model, we obtained optimized geometries for the binding of an unsaturated hydrocarbon into the monocopper cation, as shown in Figure 2.

Figure 2 shows that the unsaturated hydrocarbons bind into the monocopper cation in a *η*^2^-bridging fashion. The Cu–C bond lengths were optimized to be 1.925 and 1.967 Å in the acethylene and ethylene cases, respectively. The optimized structures lie 33 kcal/mol below their dissociation limits toward an unsaturated hydrocarbon and the small zeolite model. The *E*_stabilization_ values are consistent with those reported in the previous theoretical studies [49,50,51,52]. The stabilization mainly comes from in-phase interactions between the *d*_xz_(Cu) orbital of the extraframework monocopper and a π* orbital of C_2_H_2_ or C_2_H_4_, as shown in Figure 2. Due to the orbital interactions, the Cu(I) cation can donate electrons to the empty π* orbital of an unstaturated hydrocarbon. Accordingly, their CC bonds are activated by the bindings: the optimized CC bond lengths in the acethylene (1.241 Å) and ethylene (1.388 Å) cases are longer than the unperturbed cases by 0.042 Å and 0.061 Å, respectively.

The adsorbed acethylene and ethylene cannot retain liner *D*_∞*h*_ and planar *D*_2*h*_ structures, respectively. The geometrical distortions change their vabrational structures. In fact, we see in Table 1 and Table 2 that calculated CC stretching vibrational frequencies in the adsorbed acethylene and ethylene are 1793.8 and 1529.4 cm^-1^, respectively [53]. The values are smaller than those of free acethylene and ethylene (2001.4 and 1645.2 cm^-1^, respectively). The significant decrease in the CC stretching vibrational frequencies is due to the CC bond activation. Furthermore, their bindings into the extraframework copper cation make CC stretching modes infrared (IR)-active, due to symmetry lowering. Note that CC stretching vibrational modes of free C_2_H_4_ and C_2_H_2_ span A_g_ and Σ_g_^+^, respectively and thus the modes are IR-inactive [54,55]. Similar symmetry lowering can be seen in the symmetric CH stretching mode in the adsorbed C_2_H_2_, and thus a new IR peak appears around 3273 cm^-1^ after the C_2_H_2_ binding into the monocopper cation [56,57,58].

**Table 1:** Calculated vibrational frequencies (cm^-1^) of CC and CH stretching modes of C_2_H_2_ before and after the binding into the small monocopper zeolite model.

		Free C_2_H_2_		C_2_H_2_ on Cu_1_-zeolite	
	Symmetry	Frequency	IR intensity ^a^	Frequency	IR intensity ^a^
C≡C stretch	Σ_g_^+^	2001.4	0.00	1793.8	1.12
C–H stretch	Σ_g_^+^	3400.0	0.00	3273.7	0.40
C–H stretch	Σ_u_^+^	3300.5	1	3204.0	1

^a^ IR intensities are given relative to that of the C–H stretching mode spanning Σ_u_^+^.

**Table 2:** Calculated vibrational frequencies (cm^-1^) of CC and CH stretching modes of C_2_H_4_ before and after the binding into the small monocopper zeolite model.

	Free C_2_H_4_			C_2_H_4_ on Cu_1_-zeolite	
	Symmetry	Frequency	IR intensity ^a^	Frequency	IR intensity ^a^
C≡C stretch	A_g_	1645.2	0.00	1529.4	0.32
C–H stretch	A_g_	3036.3	0.00	3023.9	0.00
C–H stretch	B_3g_	3089.8	0.00	3090.7	0.00
C–H stretch	B_1u_	3021.3	0.64	3017.7	0.98
C–H stretch	B_2u_	3117.5	1	3113.2	1

^a^ IR intensities are given relative to that of the C–H stretching mode spanning B_2__u_.

#### 3.1.2. Realistic Cu_1_–ZSM-5 model

In the previous section (3.1.1), we discussed how an unsaturated hydrocarbon binds into a two-coordinated copper cation by using the small zeolite model. Although we obtained a baseline of the Cu–C bindings, the information is not sufficient to fully understand inner Cu–C interactions in real Cu–ZSM-5 framework due to the variety of the copper coordination environment. The coordination environment should change *d*-splittings of an extraframework copper cation, and therefore the *d*–π* interactions are influenced by the copper sites. As a result, the coordination environment should determine the attraction forces operating between the copper cation and an unsaturated hydrocarbon.

**Figure 3:**
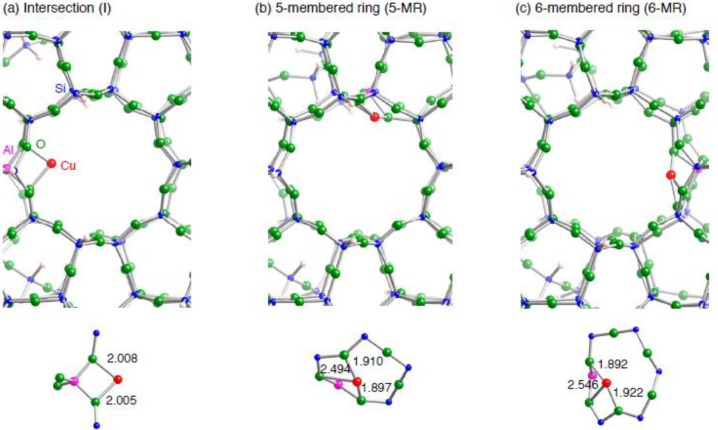
Local structures of three optimized structures for a monocopper cation sitting inside the AlSi_91_O_151_H_66_ ZSM-5 model. (a) the copper cation sits at the intersection between a straight and a zigzag channel, denoted by **I**. (b) the copper cation sits above a 5-membered ring of a wall along a straight channel, denoted by **5-MR**. (c) the copper cation sits above a 6-membered ring of a wall along a straight channel, denoted by **6-MR**.

ZSM-5 zeolite has 12 distinguishable tetrahedral(T)-sites in the orthogonal structure. Ref. [37] shows there is no significant difference between the relative energies of ZSM-5 where one Al atom replaces one Si atom in different T-sites. In addition, Nachtigall and Bell separately investigated CO adsorption into Cu–ZSM-5 with one substituted Al atom in different T-sites. Their extensive studies show that the interaction energies between CO and Cu–ZSM-5 as well as CO stretching frequencies change significantly, depending on Cu site types. Also they indicated that the location of the Al atom does not have influence on the binding energies. Judging from the interaction energies, binding sites for an extraframework cation inside ZSM-5 are categorized into three subgroups in Figure 3: one is a cation site near an intersection between a straight and a sinusoidal channels, denoted by **I**, and the others are cations located above a 5-membered and 6-membered rings of a wall along a straight channel, denoted by **5-MR** and **6-MR**, respectively. Along their theoretical findings, we considered the three binding sites for the Cu cation inside ZSM-5, where the substituted Al atom is located near the cation.

Figure 3 shows that the copper coordination environment in the **I** configuration is similar to that in the small zeolite model: in the **I** configuration, the monocopper cation is bound to two framework oxygen atoms near the substituted Al atom. In contrast, the **5-MR** and **6-MR** configurations have the monocopper cations with a coordination number of 3 [16]. Due to the different copper coordination environment, their electronic configurations are different, as shown in Table 3. The coordination of a Cu(I) cation into ZSM-5 framework results in some degree of the 3*d*^10^→3*d*^9^4*s*^1^ promotion [41]. In terms of the 3*d*^10^–3*d*^9^4*s*^1^ promotion, there is a slight difference between the **I** and **5-MR** (**6-MR**) configurations: the amount of the 4*s* electron in the **I** configuration (0.25e) is less significant than those in the **5-MR** and **6-MR** configurations (~0.4 e). Since we found the differences in the Cu coordination environments in the three configurations, it is interesting to investigate how the copper coordination environment affects the interactions with an unsaturated hydrocarbon.

**Table 3:** Electronic configurations of Cu–ZSM-5 before and after the binding of an unsaturated hydrocarbon, based on natural atomic orbital analyses (NPA).

Configuration	Adsorbent	Electronic configuration
**I**	–	4*s* ( 0.25) 3*d* ( 9.84)
	C_2_H_2_	4*s* ( 0.36) 3*d* ( 9.56) 4*p* ( 0.01)
	C_2_H_4_	4*s* ( 0.37) 3*d* ( 9.57) 4*p* ( 0.01)
**5-MR**	–	4*s* ( 0.42) 3*d* ( 9.73)
	C_2_H_2_	4*s* ( 0.36) 3*d* ( 9.57) 4*p* ( 0.01)
	C_2_H_4_	4*s* ( 0.36) 3*d* ( 9.58) 4*p* ( 0.01)
**6-MR**	–	4*s* ( 0.44) 3*d* ( 9.71)
	C_2_H_2_	4*s* ( 0.36) 3*d* ( 9.55) 4*p* ( 0.01)
	C_2_H_4_	4*s* ( 0.37) 3*d* ( 9.57) 4*p* ( 0.01)

**Figure 4:**
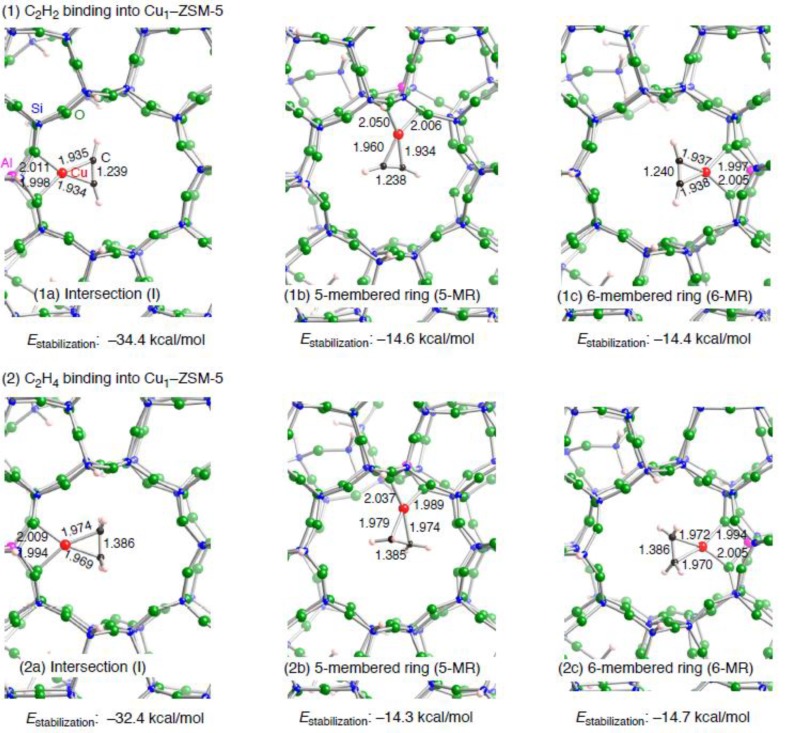
Local structures of three optimized structures for the binding of an unsaturated hydrocarbon ((1) C_2_H_2_ or (2) C_2_H_4_) into a monocopper cation embedded inside the AlSi_91_O_151_H_66_ ZSM-5 model. (a) the copper cation sits at the intersection between a straight and a zigzag channel, denoted by **I**. (b) the copper cation sits above a 5-membered ring of a wall along a straight channel, denoted by **5-MR**. (c) the copper cation sits above a 6-membered ring of a wall along a straight channel, denoted by **6-MR**. Optimized bond lengths are in Å. The *E*_stabilization_ values are in kcal/mol.

Optimized structures for unsaturated hydrocarbons adsorbed on a Cu_1_–ZSM-5 model are shown in Figure 4. Table 4 tabulates key parameters for all optimized geometries. We can see in Figure 4 the same types of bindings of the unsaturated hydrocarbons into the copper cation (*η*^2^-fashion), irrespective of different copper coordination environment as well as types of unsaturated hydrocarbon considered. Similar binding fashions have been already reported in Refs. [49,50,51,52]. In the *η*^2^-fashion of the C_2_H_2_ (C_2_H_4_) bindings, the optimized Cu–C and CC bond lengths are ~1.94 (~1.97) Å and ~1.24 (~1.38) Å, respectively. The CC bonds in the adsorbed unsaturated hydrocarbons are lengthened relative to the free unsaturated hydrocarbons, indicating that the Cu–C bond formation results in the CC bond activation. Interestingly the Cu–C bondings in the *η*^2^-fashion are completely identical to those of the small model. Note that the copper coordination environments in the three configurations are also same after the bindings, in contrast to those before the bindings. Reflecting the same Cu coordination environments, the three models have similar electron configurations of the Cu(I) cation in Table 3. In addition, the amounts of electron transferring (~0.2e) upon the bindings are similar among the three configurations. However, we see slight difference between the **I** and **5-MR** (**6-MR**) configurations in terms of how the electrons transfer between an unsaturated hydrocarbon and Cu–ZSM-5. In the **I** configuration, the 4*s* electron densities increase, whereas the 3*d* electron densities decrease. Since the two Cu–O bond lengths remain almost unchanged during the binding (Figure 3 and Figure 4), the electron transfer is responsible for the Cu–C bond formation. On the other hand, we can see that both 3*d* and 4*s* orbitals are depopulated upon the bindings in the **5-MR** and **6-MR** configurations. Compared with the **I** configuration, the electron transfers in these configurations originate from not only the formation of the two Cu–O bonds but also significant changes in the copper coordination environments. In fact we see in Figure 3 and Figure 4 that the Cu coordination number changes from 3 to 2, upon the bindings of an unsaturated hydrocarbon into an extraframework copper at **5-MR** or **6-MR** configuration. In contract, such changes in the Cu coordination number are not seen in the **I** configuration.

Despite the same binding types in the three configurations, the stabilization energies depend on their copper coordination environment: the calculated *E*_stabilization_ values in the **I** configuration (–34.4 (C_2_H_2_) and –32.4 (C_2_H_4_) kcal/mol) are more pronounced than those in the **5-MR** configuration (–14.6 (C_2_H_2_) and –14.3 (C_2_H_4_) kcal/mol) and the **6-MR** configuration (–14.4 (C_2_H_2_) and –14.7 (C_2_H_4_) kcal/mol). The stabilization energies in the **I** configuration are almost the same as those in the small zeolite models, whereas those in the **5-MR** and **6-MR** configurations are different. The different *E*_stabilization_ values are also associated with the changes in the copper coordination environments upon the bindings. Here we consider quantitatively why the copper coordination environment plays an essential role in determining the stabilization energies. In general, an extraframework cation shifts from its original position by approaching of an adsorbent to the cation. The cation shifts destabilize a zeolite structure itself. The destabilization by the cation shifts counteracts direct attractive interactions between an extraframework cation and an adsorbent. Thus the balance between the destabilization by the cation shifts and the stabilization by the direct interactions determines a stable conformation of an adsorbent inside a zeolite. The importance of the balance has been already discussed by Nachtigall and coworkers [59,60]. We can see dependences of the destabilization on the copper coordination environment in Table 4, where the destabilization energies *E*(deform) are listed. The *E*(deform) values are defined by *E*(deformed Cu–ZSM-5) – *E*(adsorbent-free Cu–ZSM-5). Here *E*(deformed Cu–ZSM-5) is the single-point-energy of a deformed Cu–ZSM-5 taken from an optimized adsorbent–Cu–ZSM-5 complex, and *E*(adsorbent-free Cu–ZSM-5) is the total energy of an optimized adsorbent-free Cu–ZSM-5 structure. Positive *E*(deform) values indicate that a Cu–ZSM-5 itself is destabilized by the inner coordination bond formation. Table 4 indicates strong site-dependencies of the *E*(deform) values.

**Table 4:** Key parameters of the optimized structures for an unsaturated hydrocarbon adsorbed on a dicopper active center embedded inside a realistic ZSM-5 model.

Configuration ^a^	Adsorbent	Binding mode	Cu–C^b^	CC^b^	*E*_stabilization_^c^	*E*(deform) ^d^
**I**	C_2_H_2_	*η*^2^	1.934, 1.935	1.239	–34.4	2.6
**5MR**	C_2_H_2_	*η*^2^	1.934, 1.960	1.238	–14.6	20.4
**6MR**	C_2_H_2_	*η*^2^	1.937, 1.938	1.240	–14.4	22.5
**I**	C_2_H_4_	*η*^2^	1.969, 1.974	1.386	–32.4	2.3
**5MR**	C_2_H_4_	*η*^2^	1.974, 1.979	1.385	–14.3	17.8
**6MR**	C_2_H_4_	*η*^2^	1.970, 1.972	1.386	–14.7	20.3

^a^ Configuration: I is the intersection site, 5MR and 6MR are the 5- and 6-membered sites.; ^b^ Bond lengths in Å.; ^c^ *E*_stabilization_ in kcal/mol.; ^d^ *E*(deform) in kcal/mol.

In fact the *E*(deform) values in the **I** configuration (2.6 (C_2_H_2_) and 2.3 (C_2_H_4_) kcal/mol) are negligible relative to those in the **5-MR** configuration (20.4 (C_2_H_2_) and 17.8 (C_2_H_4_) kcal/mol) and the **6-MR** configuration (22.5 (C_2_H_2_) and 20.3 (C_2_H_4_) kcal/mol). The site-dependent *E*(deform) values are reasonable, because decreasing the copper coordination number in the **5-MR** and **6-MR** configurations loses attractive Cu–O interactions at some extent. Taking the different *E*(deform) values into account, we can understand that bindings of an unsaturated hydrocarbon to the monocopper cation in the **I** configurations are energetically favorable over those in the **5-MR** and **6-MR** configurations. Note that the deformation energies are more significant than those in the interaction with NO molecule (the *E*(deform) values are 1 and 8 kcal/mol for intersection and channel wall sites, respectively [41]). The larger *E*(deform) values suggest that the *η*^2^-bindings require larger displacement of copper cations rather than the *η*^1^-bindings. In this situation we demonstrated from DFT calculations that the copper coordination environment is a key factor determining the bindings of an unsaturated hydrocarbon into an extraframework monocopper cation of ZSM-5.

### 3.2. ZSM-5 containing dicopper active center (Cu_2_–ZSM-5)

In this section we will focus on an unsaturated hydrocarbon bound to a dicopper active center embedded in ZSM-5. In this situation, configurations of the two copper cations within a ZSM-5 cavity may be responsible for the unsaturated hydrocarbon bindings, in addition to their copper coordination environment. Experimentally the presence of Cu pairs in ZSM-5 was demonstrated by using extended X-ray absorption fine structure (EXAFS) [61,62,63] spectroscopy and X-ray power diffraction [45] studies. To fully understand behaviors of an unsaturated hydrocarbon inside a ZSM-5 cavity, it is indispensable to clarify how configurations of the two copper cations inside ZSM-5 affect the properties of adsorbed unsaturated hydrocarbons.

#### 3.2.1. The small dicopper zeolite model

**Figure 5:**
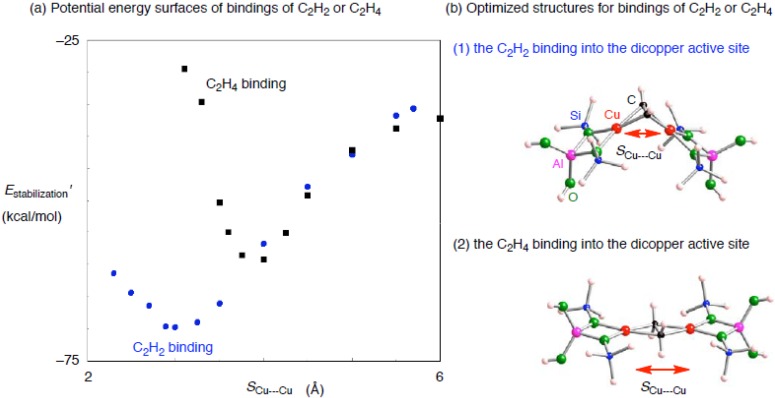
(a) Potential energy surfaces of approaching of an unsaturated hydrocarbon (C_2_H_2_ or C_2_H_4_) into the smaller dicopper zeolite model ([Cu–Al_1_Si_3_O_4_H_8_]_2_). *E*_stabilization_’ is plotted as a function of the Cu•••Cu separation. (b) optimized structures for the binding of an unsaturated hydrocarbon ((1) C_2_H_2_ or (2) C_2_H_4_) into the small dicopper zeolite model.

First we use a small zeolite model containing the dicopper active center ([Cu–AlSi_2_O_4_H_8_]_2_) to increase our preliminary understanding of the interactions with an unsaturated hydrocarbon. Using the small dicopper zeolite model, we optimized an unsaturated hydrocarbon adsorbed on the dicopper active center, except for the Cu•••Cu separation (*S*_Cu•••Cu_). Potential energy surfaces of the approaching of an unsaturated hydrocarbon into the dicopper active center are seen in Figure 5 as a function of *S*_Cu•••Cu_. Figure 5 shows their stabilization energies *E*_stabilization_’ defined as – *E*(adsorbent) – *E*(Cu_2_–zeolite[*S*_Cu•••Cu_]) + *E*(adsorbent–Cu_2_–zeolite[*S*_Cu•••Cu_]), where *E*(adsorbent) is the total energy of the optimized structure for an adsorbent, *E*(adsorbent–Cu_2_–zeolite[*S*_Cu•••Cu_]) is that of an optimized structure for an adsorbent bound to the small dicopper zeolite at a certain *S*_Cu•••Cu_ value, and *E*(Cu_2_–zeolite[*S*_Cu•••Cu_]) is that of a Cu_2_–zeolite taken from the optimized adsorbent–Cu_2_–zeolite[*S*_Cu•••Cu_] structure. When *S*_Cu•••Cu_ is 6 Å, C_2_H_2_ or C_2_H_4_ binds into one copper cation bound to the AlSi_2_O_4_H_8_ zeolite model. Then the optimized structures for the C_2_H_2_ and C_2_H_4_ bindings are, respectively, 35.6 and 37.2 kcal/mol stable relative to those in the dissociation limits toward an unsaturated hydrocarbon and the free small zeolite model with the two copper cations apart by 6 Å. Of course, the *E*_stabilization_’ values are close to those obtained in the small monocopper zeolite.

Decreasing the Cu•••Cu separation, two Cu–C bonds are newly generated. As a result, an unsaturated hydrocarbon binds into both copper cations in a *μ-η*^2^: *η*^2^ fashion. Because of the new Cu–C bond formation, the *E*_stabilization_’ values lower significantly until a certain *S*_Cu•••Cu_ value. In fact we see in Figure 5 that the potential energy surface of the C_2_H_2_–dicopper (C_2_H_4_–dicopper) complex has one local minimum at *S*_Cu•••Cu_ of 2.888 (3.735) Å.

As shown in Figure 5 and Table 5 the optimized C_2_H_2_– and C_2_H_4_–dicopper complexes lie, respectively, 34.0 and 21.4 kcal/mol below the structures with the *S*_Cu•••Cu_ values of ~6 Å. These DFT results clearly show that the interactions of acethylene with the dicopper active center are more significant than the ethylene case. The differences between the acethylene and ethylene additions are unique in the dicopper cases, which cannot be seen in the monocopper cases. Moreover, we can see some discrepancy in the optimized structures between the acathylene and ethylene cases: the optimized C_2_H_2_–Cu_2_–zeolite contains a *μ-η*^2^: *η*^2^ Cu_2_C_2_ core with a butterfly form, whereas the C_2_H_4_–Cu_2_–zeolite contains a planar *μ-η*^2^: *η*^2^ core. Reflecting the structural differences, the C_2_H_2_–Cu_2_–zeolite has a smaller *S*_Cu•••Cu_ value than that in C_2_H_4_–Cu_2_–zeolite.

**Table 5:** Key parameters of the optimized structures of an unsaturated hydrocarbon adsorbed onto a dicopper active center bound to a small zeolite model.

Adsorbent	Binding mode	Cu–C^a^	CC^a^	*S*_Cu•••Cu_^b^	*E*_stabilization_’^c^
C_2_H_2_	*μ−η*^2^: *η*^2^	1.932, 1.933 1.933, 1.934	1.297	2.888	–69.6
C_2_H_4_	*μ−η*^2^: *η*^2^	2.003, 2.003, 2.003, 2.003	1.449	3.735	–58.6

^a^ Bond lengths in Å.; ^b^*S*_Cu•••Cu_ is the Cu•••Cu separation in Å.; ^c^ *E*_stabilization_’ in kcal/mol.

Whether the Cu_2_C_2_ core has a planar or a butterfly structure can be confirmed by their IR spectra, especially their CC stretching vibrational modes (Table 6 and Table 7). We see in Table 6 and Table 7 that CC stretching vibrational frequencies were calculated to be 1557.1 and 1469.4 cm^-1^ in C_2_H_2_ and C_2_H_4_ adsorbed on the dicopper active center, respectively. Lower CC stretching frequencies than those in the monocopper model are ascribed to more significant CC bond activation by the dicopper active center: the optimized CC bond lengths in the acethylene and ethylene additions are 1.297 and 1.449 Å, respectively (see Table 5). More importantly, the CC stretching vibrational mode in the acethylene addition is IR-active, whereas that in the ethylene addition is IR-inactive due to the planarity of its Cu_2_C_2_ core. The calculated IR data will help to determine how an unsaturated hydrocarbon binds into a dicopper active center embedded inside ZSM-5.

**Table 6:** Calculated vibrational frequencies (cm^-1^) of CC and CH stretching modes of C_2_H_2_ before and after the binding into the small dicopper zeolite model.

	Free C_2_H_2_			C_2_H_2_ on Cu_2_-zeolite	
	Symmetry	Frequency	IR intensity^a^	Frequency	IR intensity^a^
C≡C stretch	Σ_g_^+^	2001.4	0.00	1557.1	1.44
C–H stretch	Σ_g_^+^	3400.0	0.00	3170.0	0.90
C–H stretch	Σ_u_^+^	3300.5	1	3127.8	1

^a^ IR intensities are given relative to that of the C–H stretching mode spanning Σ_u_^+^.

**Table 7:** Calculated vibrational frequencies (cm^-1^) of CC and CH stretching modes of C_2_H_4_ before and after the binding into the small dicopper zeolite model.

	Free C_2_H_4_			C_2_H_4_ on Cu_2_-zeolite	
	Symmetry	Frequency	IR intensity ^a^	Frequency	IR intensity ^a^
C≡C stretch	A_g_	1645.2	0.00	1469.4	0.00
C–H stretch	A_g_	3036.3	0.00	3004.9	0.00
C–H stretch	B_3g_	3089.8	0.00	3085.4	0.00
C–H stretch	B_1u_	3021.3	0.64	3001.8	17.10
C–H stretch	B_2u_	3117.5	1	3100.5	1

^a^ IR intensities are given relative to that of the C–H stretching mode spanning Β_2__u_.

#### 3.2.2. Realistic Cu_2_–ZSM-5 model

**Figure 6:**
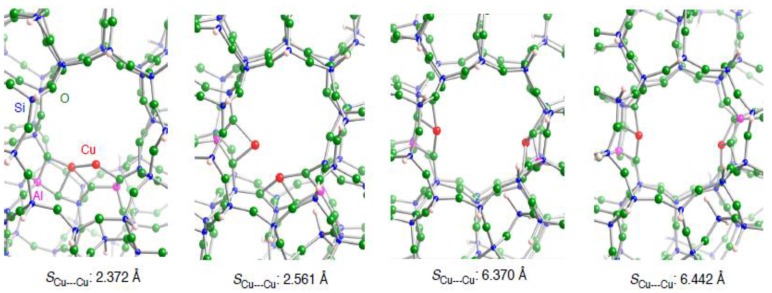
Local structures of four optimized Cu_2_–ZSM-5 structures where the ZSM-5 framework was modeled as a Al_2_Si_90_O_151_H_66_ cluster. The four types of optimized structures can be distinguished by positions of double Si→Al substitution: the two substituted Al atoms within a ten-membered ring are second nearest neighbor (**2NN**), third nearest neighbor (**3NN**), fourth nearest neighbor (**4NN**), and fifth nearest neighbor (**5NN**). Optimized bond lengths are given in Å.

In section 3.2.1, we used the small dicopper zeolite model, and found differences between the dicopper and monocopper cations in terms of the interactions with an unsaturated hydrocarbon. Next we turn to dicopper active centers located in a 10-MR cavity of the realistic ZSM-5 model Al_2_Si_90_O_151_H_60_. Since copper cations usually sit near the Al substituted positions, the locations of the double Si→Al substitution within the ZSM-5 framework control the configurations of a dicopper active center. Here we consider four locations of the double substitution in Figure 6: the **2NN**, **3NN**, **4NN**, and **5NN** configurations contain the Al pairs being, respectively second, third, fourth, and fifth nearest-neighbors with respect to tetrahedral sites contained in the 10-MR. Using the different configurations of the Al pair, their initial geometries were constructed by placing each Cu apart by ~2.0 Å from two oxygen atoms bound to a substituted Al atom. After the B3LYP optimization, four configurations of the dicopper center inside ZSM-5 were obtained, as shown in Figure 6. The Cu•••Cu separation in Cu_2_–ZSM-5 decreases in the order **5NN** (6.442 Å) > **4NN** (6.370 Å) > **3NN** (2.561 Å) > **2NN** (2.372 Å). The Cu•••Cu separations in the **2NN** and **3NN** configurations are close to those obtained by EXAFS analyses (2.47–3.13 Å). In the optimized Cu_2_–ZSM-5 structures, each Cu cation coordinates into two or three framework oxygen atoms. The Cu•••Cu separations are out of the range of a suitable span between the two copper cations into which an unsaturated hydrocarbon preferentially binds in a μ-*η*^2^: *η*^2^-fashion (Figure 5). Note that the formation of Cu pairs in the **3NN** and **2NN** configurations is consistent with the Spuhler’s findings [38] by means of a combined quantum mechanics/interatomic potential function technique(QM-pot).

**Figure 7:**
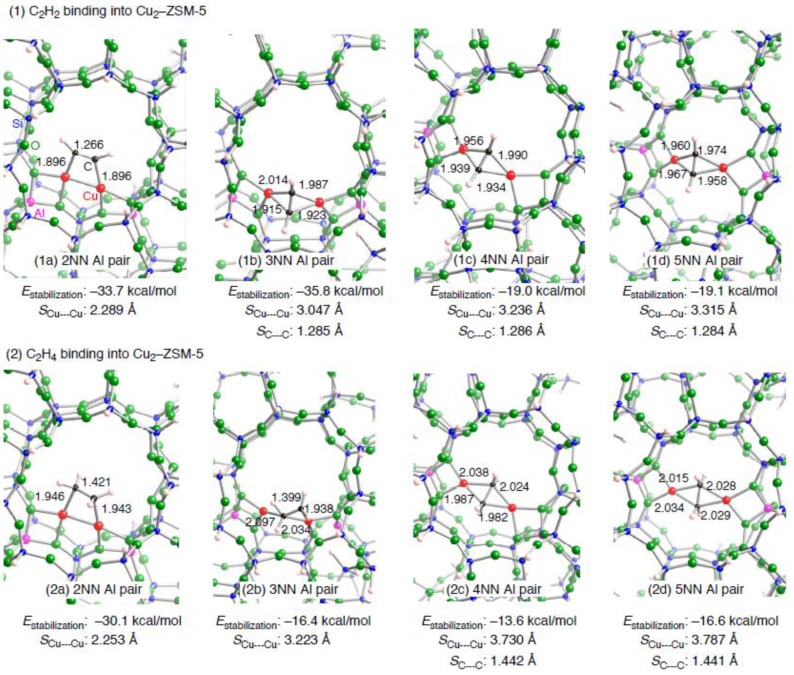
Local structures of four optimized structures for the binding of an unsaturated hydrocarbon ((1) C_2_H_2_ or (2) C_2_H_4_) into a dicopper active center embedded inside the Al_2_Si_90_O_151_H_66_ ZSM-5 model. The four types of optimized structure can be distinguished by positions of double Si→Al substitution: the two substituted Al atoms within a ten-membered ring are (a) second nearest neighbor (**2NN**), (b) third nearest neighbor (**3NN**), (c) fourth nearest neighbor (**4NN**), and fifth nearest neighbor (**5NN**). Optimized bond lengths together with the Cu•••Cu separations are given in Å. The *E*_stabilization_ values are in kcal/mol.

Taking varying Cu•••Cu separations by locations of the double Si→Al substitution into account, we discuss how an unsaturated hydrocarbon binds into a dicopper active center. Figure 7 shows the optimized structures for an unsaturated hydrocarbon adsorbed on a dicopper active center embedded in a ZSM-5 model, whose key parameters are listed in Table 8. We can see in Figure 7 and Table 8 two types of binding of C_2_H_2_ into a dicopper active center. In the **2NN** configuration, C_2_H_2_ binds into the dicopper active center in a *μ*-*η*^1^: *η*^1^ fashion, whereas the **3NN**, **4NN**, and **5NN** configurations have a *μ*-*η*^2^: *η*^2^ Cu_2_C_2_ core. In the **3NN**, **4NN**, and **5NN** configurations, the Cu•••Cu separations were optimized to be 3.047, 3.236, and 3.315 Å, respectively. These *S*_Cu•••Cu_ values are close to the separation between the two copper cations into which C_2_H_2_ binds in a *μ*-*η*^2^: *η*^2^ fashion in Figure 5. The significant differences in the *S*_Cu•••Cu_ values between before and after the C_2_H_2_ binding (*ΔS*_Cu•••Cu_) indicate that two copper cations significantly shift after the binding. In contrast, the **2NN** configuration does not have a room, and thus the two copper cations cannot move to positions suitable for the C_2_H_2_ binding in a *μ*-*η*^2^: *η*^2^ fashion. Instead, the **2NN** configuration adopts the *μ*-*η*^1^:*η*^1^ binding fashion with a stabilization energy of –33.7 kcal/mol. Surprisingly the *E*_stabilization_ value is comparable to that of the **3NN** configuration (–35.8 kcal/mol), despite the different C_2_H_2_ binding fashions.

In the above discussion, we found that whether the two copper cations smoothly shift determines a preferable C_2_H_2_ binding fashion in a ZSM-5 cavity. Although the importance of the copper shifts can be also seen in the monocopper case, the *E*(deform) values in the dicopper cases (Table 8) are more pronounced than those in the monocopper cases. In addition, we found from Table 8 strong dependences of the *E*(deform) values on the dicopper configurations: the *E*(deform) values decrease in the following order: **5NN** ≈ **4NN** > **3NN** > **2NN**. The decrease in the *E*(deform) values has a close relation to the *ΔS*_Cu•••Cu_ values. Table 8 shows that the *ΔS*_Cu•••Cu_ values decrease in the order **4NN** ≈ **5NN** > **3NN** > **2NN**, suggesting that the C_2_H_2_ bindings in the **5NN** and **4NN** configurations cause two copper cations to shift significantly, compared with those in the **3NN** and **2NN** configurations. Therefore, the dependent *E*(deform) values are understandable. Reflecting the *E*(deform) values, the stabilization in the **4NN** and **5NN** configurations is less significant than that in the **3NN** configuration, because the destabilization by the cation shifts diminishes the attraction by the direct interactions. Thus the variety of the Cu–C bonding characters is unique in the restricted environment of ZSM-5.

**Table 8:** Key parameters of the optimized structures of an unsaturated hydrocarbon adsorbed onto a dicopper active center embedded inside a realistic ZSM-5 model.

Configuration ^a^	Adsorbent	Binding mode	Cu–C ^b^	CC ^b^	*S*_Cu•••Cu_^c^ (Δ*S*_Cu•••Cu_^d^)	*E*_stabilization_^e^	*E*(deform) ^f^
**2NN**	C_2_H_2_	*μ−η*^1^: *η*^1^	1.896, 1,896	1.266	2.289 (–0.083)	–33.7	19.0
**3NN**	C_2_H_2_	*μ−η*^2^: *η*^2^	1.915, 1.923, 1.987, 2.014	1.285	3.047 (0.486)	–35.8	24.7
**4NN**	C_2_H_2_	*μ−η*^2^: *η*^2^	1.934, 1.939, 1.956, 1.990	1.286	3.236 (–3.134)	–19.0	45.7
**5NN**	C_2_H_2_	*μ−η*^2^: *η*^2^	1.958, 1.960, 1.967, 1.974	1.284	3.315 (–3.127)	–19.1	47.2
**2NN**	C_2_H_4_	*μ−η*^1^: *η*^1^	1.946, 1.943	1.421	2.253 (–0.119)	–30.1	18.0
**3NN**	C_2_H_4_	*μ−η*^1^: *η*^2^	1.938, 2.034, 2.097	1.399	3.223 (0.662)	–16.4	25.5
**4NN**	C_2_H_4_	*μ−η*^2^: *η*^2^	1.982, 1.987, 2.024, 2.038	1.442	3.730 (–2.640)	–13.6	41.9
**5NN**	C_2_H_4_	*μ−η*^2^: *η*^2^	2.015, 2.028, 2.029, 2.034	1.441	3.787 (–2.655)	–16.6	43.1

^a^ Configuration: The **2NN**, **3NN**, and **4NN** configurations have Al pairs being second-, third-, and fourth nearest neighbors within a ten-membered ring of the ZSM-5 model, respectively.; ^b^ Bond lengths in Å.; ^c^ *S*_Cu•••Cu_ is the Cu•••Cu separation in Å.; ^d^ Δ*S*_Cu•••Cu_ is the difference in the Cu•••Cu separation between before and after the binding of an unsaturate hydrocarbon.; ^e^ *E*_stabilization_’ in kcal/mol.; ^f^ *E*(deform) in kcal/mol.

Compared with the C_2_H_2_ bindings, more complicated binding modes are found in the C_2_H_4_ bindings into a dicopper center in Figure 7: the **2NN** configuration has a *μ*-*η*^1^:*η*^1^ form*,* the **3NN** configuration has a *μ*-*η*^1^:*η*^2^ form, and the **4NN** and **5NN** configurations have a *μ*-*η*^2^:*η*^2^ form. The calculated *E*_stabilization_ values are close to those obtained experimentally (–15.4 and –21.4 kcal/mol) [64]. The *μ*-*η*^2^:*η*^2^ binding fashions in the **4NN** and **5NN** configurations are similar to that obtained in the small zeolite model: in both cases, the Cu and C atoms virtually lie in a plane. The Cu•••Cu separations in the **4NN** and **5NN** configurations (3.730 and 3.787 Å, respectively) are essentially identical to the equilibrium separation between C_2_H_4_ and the small zeolite model (3.735 Å in Figure 5), and thus the similarity in the binding fashion is reasonable. In contrast, the optimized *S*_Cu•••Cu_ values in the **2NN** and **3NN** configurations are 1.482 and 0.512 Å smaller than the equilibrium separation in the small dicopper zeolite model. Accordingly these configurations cannot adopt a *μ*-*η*^2^:*η*^2^ binding fashion. Note that the *μ*-*η*^1^:*η*^2^ binding fashion (**3NN**) is an intermediate between the *μ*-*η*^1^:*η*^1^ (**2NN**) and *μ*-*η*^2^:*η*^2^ (**4NN** and **5NN**) fashions. How C_2_H_4_ binds into a dicopper active center inside ZSM-5 is also followed by the balance rule. As shown in Table 8, destabilization of the Cu_2_–ZSM-5 by the C_2_H_4_ binding is similar to that by the C_2_H_2_ binding form a viewpoint of energetics. However, direct interactions by the C_2_H_4_ binding are 11.0 kcal/mol weaker than those by the C_2_H_2_ binding (see Table 5). Compared with the C_2_H_2_ bindings, the importance of the destabilization by the cation shifts to determine a preferable C_2_H_4_ binding fashion is more effective rather than the direct attractive C_2_H_4_–dicopper interactions.

## 4. Conclusions

We found from large-scale DFT calculations that characters of copper–carbon bonds formed inside ZSM-5 change significantly, depending on its copper coordination environment. Actually attractive interactions of an unsaturated hydrocarbon with a two-coordinated extraframework copper cation are significant relative those with a higher-coordinated copper cation. The dependences of the interactions are related with shifts of a copper cation accompanied by the bindings: the shift of a cation with a higher coordination number costs energy a lot. Thus site-preferences of two-coordinated copper cations as the unsaturated-hydrocarbon binding site are reasonable. When an unsaturated hydrocarbon binds into an embedded dicopper active center, configurations of the two copper cations are important to determine the bindings, in addition to its coordination environment. Because of the different interactions between an unsaturated hydrocarbon and a mono- or dicopper copper active center, various binding fashions (*η*^2^, *μ*-*η*^1^:*η*^1^, *μ*-*η*^1^:*η*^2^, and *μ*-*η*^2^:*η*^2^ fashions) are expected in Cu–ZSM-5. The variety of characters of the copper–carbon bonds is unique in the restricted environment of a zeolite.

## References

[1] Crabtree R.H. (1994). The Organometallic Chemistry of the Transtion Metals.

[2] Crabtree R.H. (1985). The organometallic chemistry of alkanes. Chem. Rev..

[3] Bergman B.G. (2007). C–H activation. Nature.

[4] Arndtsen B.A., Bergman B.G., Mobley T.A., Peterson T.H. (1995). Selective intermolecular carbon-hydrogen bond activation by synthetic metal complexes in homogeneous solution. Acc. Chem. Res..

[5] Schröder D., Schwarz H. (1995). C-H and C-C bond activation by bare transition-metal oxide cations in the gas phase. Angew. Chem. Int. Ed. Engl..

[6] Eller K., Schwarz H. (1991). Organometallic chemistry in the gas phase. Chem. Rev..

[7] Weisshaar J.C. (1993). Bare transition metal atoms in the gas phase: reactions of M, M^+^, and M^2+^ with hydrocarbons. Acc. Chem. Res..

[8] Trost B.M. (2002). On inventing reactions for atom economy. Acc. Chem. Res..

[9] Hahn C. (2004). Enhancing electrophilic alkene activation by increasing the positive net charge in transition-metal complexes and application in homogeneous catalysis. Chem. Eur. J..

[10] Shriver D.F., Atkins P.W. (2006). Inorganic Chemistry.

[11] Yumura T., Kertesz M., Iijima S. (2007). Local modifications of single-wall carbon nanotubes induced by bond formation with encapsulated fullerenes. J. Phys. Chem. B.

[12] Yumura T., Kertesz M. (2007). Cooperative behaviors in carbene additions through local modifications of nanotube surfaces. Chem. Mater..

[13] Yumura T., Kertesz M., Iijima S. (2007). Confinement effects on site-preferences for cycloadditions into carbon nanotubes. Chem. Phys. Lett..

[14] Yumura T., Takeuchi M., Kobayashi H., Kuroda Y. (2009). Effects of ZSM-5 zeolite confinement on reaction intermediates during dioxygen activation by enclosed dicopper cations. Inorg. Chem..

[15] Itadani A., Sugiyama H., Tanaka M., Ohkubo T., Yumura T., Kobayashi H., Kuroda Y. (2009). Potential for C−H Activation in CH_4_ Utilizing a CuMFI-Type Zeolite as a Catalyst. J. Phys. Chem. C.

[16] Yumura T., Yamashita H., Torigoe H., Kobayashi H., Kuroda Y. (2010). Site-specific Xe addition into Cu–ZSM-5 zeolite. Phys. Chem. Chem. Phys..

[17] Iwamoto M., Furukawa H., Mine Y., Uemura F., Mikuriya S., Kagawa S. (1986). Copper(II) ion-exchanged ZSM-5 zeolites as highly active catalysts for direct and continuous decomposition of nitrogen monoxide. J. Chem. Soc., Chem. Commun..

[18] Kuhn P., Pale P., Sommer J., Louis B. (2009). Probing Cu-USY zeolite reactivity: design of a green catalyst for the synthesis of diynes. J. Phys. Chem. C.

[19] Yu J.-S., Kevan L. (1991). Effects of reoxidation and water vapor on selective partial oxidation of propylene to acrolein in copper(II)-exchanged X and Y zeolites. J. Phys. Chem..

[20] Becke A.D. (1988). Density-functional exchange-energy approximation with correct asymptotic behavior. Phys. Rev. A.

[21] Becke A.D. (1993). Density-functional thermochemistry. III. The role of exact exchange. J. Chem. Phys..

[22] Stephens P.J., Devlin F.J., Chabalowski C.F., Frisch M.J. (1994). Ab initio calculation of vibrational absorption and circular dichroism spectra using density functional force fields. J. Phy. Chem..

[23] Lee C., Yang W., Parr R.G. (1988). Development of the Colle-Salvetti correlation-energy formula into a functional of the electron density. Phys. Rev. B.

[24] Vosko S.H., Wilk L., Nusair M. (1980). Accurate spin-dependent electron liquid correlation energies for local spin density calculations: a critical analysis. Can. J. Phys..

[25] Frisch M.J., Trucks G.W., Schlegel H.B., Scuseria G.E., Robb M.A., Cheeseman J.R., Montgomery J.A., Vreven T., Kudin K.N., Burant J.C. (2003). Gaussian 03.

[26] (2001). The ZSM-5 structure was taken from the Cerius 2 database.

[27] Krishnan R., Binkley J.S., Seeger R., Pople J.A. (1980). Self-consistent molecular orbital methods. XX. A basis set for correlated wave functions. J. Chem. Phys..

[28] Wachters A.J.H. (1970). Gaussian basis set for molecular wavefunctions containing third-row atoms. J. Chem. Phys..

[29] Hay R.J. (1977). Gaussian basis sets for molecular calculations. The representation of 3d orbitals in transition-metal atoms. J. Chem. Phys..

[30] Hehre W.J., Ditchfield R., Pople J.A. (1972). Self—Consistent Molecular Orbital Methods. XII. Further Extensions of Gaussian—Type Basis Sets for Use in Molecular Orbital Studies of Organic Molecules. J. Chem. Phys..

[31] Francl M.M., Pietro W.J., Hehre W.J., Binkley J.S., Gordon M.S. (1982). Self-consistent molecular orbital methods. XXIII. A polarization-type basis set for second-row elements. J. Chem. Phys..

[32] Hariharan P.C., Pople J.A. (1973). The influence of polarization functions on molecular orbital hydrogenation energies. Theor. Chim. Acta.

[33] Binkley J.S., Pople J.A., Hehre W.J. (1980). Self-consistent molecular orbital methods. 21. Small split-valence basis sets for first-row elements. J. Am. Chem. Soc..

[34] Gordon M.S., Binkley J.S., Pople J.A., Pietro W.J., Hehre W.J. (1982). Self-consistent molecular-orbital methods. 22. Small split-valence basis sets for second-row elements. J. Am. Chem. Soc..

[35] Pietro W.J., Francl M.M., Gordon M.S., Hehre W.J., Defrees D.J., Pople J.A., Binkley J.S. (1982). Self-consistent molecular orbital methods. 24. Supplemented small split-valence basis sets for second-row elements. J. Am. Chem. Soc..

[36] Boys S.F., Bernardi F. (1970). The calculation of small molecular interactions by the differences of separate total energies. Some procedures with reduced errors. Mol. Phys..

[37] Nachtigallová D., Nachtigall P., Sierka M., Sauer J. (1999). Coordination and siting of Cu^+^ ions in ZSM-5: A combined quantum mechanics/interatomic potential function study. Phys. Chem. Chem. Phys..

[38] Spuhler P., Holthausen M.C., Nachtigallová D., Nachtigall P., Sauer J. (2002). On the Existence of CuI Pairs in ZSM-5 - A Computational Study. Chem. Eur. J..

[39] Davidová M., Nachtigallová D., Bulánek R., Nachtigall P. (2003). Characterization of the Cu^+^ sites in high-silica zeolites interacting with the CO molecule: combined computational and experimental Study. J. Phys. Chem. B.

[40] Bulánek R., Silhan M., Nachtigallová D., Nachtigall P. (2003). Calculations of site-specific CO stretching frequencies for copper carbonyls with the “near apectroscopic accuracy”: CO interaction with Cu^+^/MFI. J. Phys. Chem. A.

[41] Davidová M., Nachtigallová D., Nachtigall P., Sauer J. (2004). Nature of the Cu^+^–NO Bond in the Gas Phase and at Different Types of Cu^+^ Sites in Zeolite Catalysts. J. Phys. Chem. B.

[42] Bludský O., Silhan M., Nachtigall P., Bucko T., Benco L., Hafner J. (2005). Theoretical Investigation of CO Interaction with Copper Sites in Zeolites: Periodic DFT and Hybrid Quantum Mechanical/Interatomic Potential Function Study. J. Phys. Chem. B.

[43] Bulánek R., Drobná H., Nachtigall P., Rubeš M., Bludský O. (2006). On the site-specificity of polycarbonyl complexes in Cu/zeolites: combined experimental and DFT study. Phys. Chem. Chem. Phys..

[44] Zheng X., Zhang Y., Bell A.T. (2007). Density Functional Theory Study of CO Adsorption on Cu(I)-ZSM-5. J. Phys. Chem. C.

[45] Mentzen B.F., Bergeret G. (2007). Crystallographic Determination of the Positions of the Copper Cations in Zeolite MFI. J. Phys. Chem. C.

[46] Kumashiro R., Kuroda Y., Nagao M. (1999). New analysis of oxidation state and coordination environment of copper ion-exchanged in ZSM-5 Zeolite. J. Phys. Chem. B.

[47] Kuroda Y., Kumashiro R., Yoshimoto T., Nagao M. (1999). Characterization of active sites on copper ion-exchanged ZSM-5-type zeolite for NO decomposition reaction. Phys. Chem. Chem. Phys..

[48] Kuroda Y., Yagi K., Horiguchi N., Yoshikawa Y., Kumashiro R., Nagao M. (2003). New light on the state of active sites in CuZSM-5 for the NO decomposition reaction and N_2_ adsorption. Phys. Chem. Chem. Phys..

[49] Hüber G., Rauhut G., Stoll H., Roduner E. (2003). Ethyne adsorbed on CuNaY zeolite: FTIR spectra and quantum chemical calculations. J. Phys. Chem. B.

[50] Hüber G., Rauhut G., Stoll H., Roduner E. (2002). FTIR measurements and quantum chemical calculations of Ethylene adsorbed on CuNaY. Phys. Chem. Chem. Phys..

[51] Broclawik E., Rejmak P., Kozyra P., Datka J. (2006). DFT quantum chemical modeling of the interaction of alkenes with Cu^+^ sites in zeolites. Catal. Today.

[52] Rejmak P., Mitoraj M., Broclawik E. (2010). Electronic view on ethene adsorption in Cu(I) exchanged zeolites. Phys. Chem. Chem. Phys..

[53] Halls M.D., Velkovski J., Schlegel H.B. (2001). Harmonic frequency scaling factors for Hartree-Fock, S-VWN, B-LYP, B3-LYP, B3-PW91 and MP2 with the Sadlej pVTZ electric property basis set. Theor. Chem. Acc..

[54] Herzberg G. (1991). Molecular Spectra and Molecular Structure: Infrared and Raman Spectra of Polyatomic Molecules.

[55] Willian K.R., Ewing E. (1995). Infrared spectra and structures of ethene on NaCl(100). J. Phys. Chem..

[56] Datka J., Kukulska-Zając E., Kobyzewa W. (2005). The activation of acetylene by Cu^+^ ions in zeolites studied by IR spectroscopy. Catal. Today.

[57] Datka J., Kukulska-Zając E. (2004). IR studies of the activation of C=C bond in alkenes by Cu^+^ ions in zeolites. J. Phys. Chem. B.

[58] Itadani A., Yumura T., Ohkubo T., Kobayashi H., Kuroda Y. (2010). Existence of dual species composed of Cu^+^ in CuMFI being bridged by C_2_H_2_. Phys. Chem. Chem. Phys..

[59] Nachtigallová D., Bludsky O., Areán C.O., Bulánek R., Nachtigall P. (2006). The vibrational dynamics of carbon monoxide in a confined space–CO in zeolites. Phys. Chem. Chem. Phys..

[60] Nachtigall P., Bulánek R. (2006). Theoretical investigation of site-specific characteristics of CO adsorption complexes in the Li^+^-FER zeolite. Appl. Catal., A.

[61] Palomino G.T., Fisicaro P., Bordiga S., Zecchina A., Giamello E., Lamberti C. (2000). Oxidation states of copper ions in ZSM-5 zeolites. a multitechnique investigation. J. Phys. Chem. B.

[62] Hamada H., Matsubayashi N., Shimada H., Kintaichi Y., Ito T., Nishijima A. (1990). XANES and EXAFS analysis of copper ion-exchanged ZSM-5 zeolite catalyst used for nitrogen monoxide decomposition. Catal. Lett..

[63] Grünert W., Hayes N.W., Joyner R.W., Shpiro E.S., Siddiqui M.R.H., Baeva G.N. (1994). Structure, chemistry, and activity of Cu-ZSM-5 catalysts for the selective reduction of NOx in the presence of oxygen. J. Phys. Chem..

[64] Borgard G.D., Molvik S., Balaraman P., Root T.W., Dumesic J.A. (1995). Microcalorimetric and infrared spectroscopic studies of CO, C_2_H_4_, N_2_O, and O_2_ adsorption on Cu-Y zeolite. Langmiur.

